# Absolute frequency metrology of buffer-gas-cooled molecular spectra at 1 kHz accuracy level

**DOI:** 10.1038/s41467-022-34758-9

**Published:** 2022-11-16

**Authors:** Roberto Aiello, Valentina Di Sarno, Maria Giulia Delli Santi, Maurizio De Rosa, Iolanda Ricciardi, Paolo De Natale, Luigi Santamaria, Giovanni Giusfredi, Pasquale Maddaloni

**Affiliations:** 1 Istituto Nazionale di Ottica, Consiglio Nazionale delle Ricerche, Via Campi Flegrei 34, Pozzuoli, 80078 Italy; 2 Istituto Nazionale di Fisica Nucleare, Sez. di Napoli, Complesso Universitario di M. S. Angelo, Via Cintia, Napoli, 80126 Italy; 3 Istituto Nazionale di Ottica, Consiglio Nazionale delle Ricerche, L.go E. Fermi 6, Firenze, 50125 Italy; 4 Centro di Geodesia Spaziale, Agenzia Spaziale Italiana, Contrada Terlecchia, Matera, 75100 Italy; 5 ppqSense S.r.l., Via Gattinella 20, Campi Bisenzio, 50013 Italy

**Keywords:** Atomic and molecular interactions with photons, Optics and photonics

## Abstract

By reducing both the internal and translational temperature of any species down to a few kelvins, the buffer-gas-cooling (BGC) technique has the potential to dramatically improve the quality of ro-vibrational molecular spectra, thus offering unique opportunities for transition frequency measurements with unprecedented accuracy. However, the difficulty in integrating metrological-grade spectroscopic tools into bulky cryogenic equipment has hitherto prevented from approaching the kHz level even in the best cases. Here, we overcome this drawback by an original opto-mechanical scheme which, effectively coupling a Lamb-dip saturated-absorption cavity ring-down spectrometer to a BGC source, allows us to determine the absolute frequency of the acetylene (*ν*_1_ + *ν*_3_) R(1)e transition at 6561.0941 cm^−1^ with a fractional uncertainty as low as 6 × 10^−12^. By improving the previous record with buffer-gas-cooled molecules by one order of magnitude, our approach paves the way for a number of ultra-precise low-temperature spectroscopic studies, aimed at both fundamental Physics tests and optimized laser cooling strategies.

## Abstract (web summary)

High-resolution molecular spectroscopy with cryogenic setups is hampered by the lack of a skilled interrogation tool. Here, the authors demonstrate absolute metrology of cold rovibrational spectra at 1 kHz accuracy level, by coupling a Lamb-dip saturated-absorption cavity ring-down spectrometer to a buffer-gas cooling source.

## Introduction

Vibrational and rotational molecular spectra are the target of several fundamental Physics experiments relying on ultra-precise frequency measurements^1^. These include refined Quantum Electro-Dynamics (QED) tests^2–4^, detection of parity-violating effects^5–7^, investigation of hypothetical extra forces and dimensions^8^, assessment of the time stability of fundamental constants^9,10^, and identification of symmetry-violating electromagnetic moments^11^.

Towards such ambitious goals, in addition to the most sophisticated probe laser sources and interrogation techniques, a powerful tool is now represented by the buffer-gas-cooling (BGC) technology for producing gas molecular samples at cryogenic temperatures, down to a few kelvins^12,13^. Indeed, according to the Boltzmann statistics, cooling of rotational temperatures reduces the number of populated internal states, thus suppressing spectral congestion and eventually increasing the total number of detected molecules (if the investigated transition starts from the ground state or from one energetically close to it). Simultaneously, cooling the external degrees of freedom restricts the distribution of velocities in the sample, resulting in narrower line-shapes, and raising the matter-radiation interaction time. Moreover, when using specimens in form of beams, spectra are also freed from collisional broadening effects.

Actually, up to now, these favorable properties paid off most in the case of large-size species or even complex chemical mixtures, where the drastic simplification of the spectra plays the most important role, allowing probing molecular structure and dynamics with unprecedented spectral resolution and specificity^14,15^. In this respect, a bright example is given by the observation of quantum state-resolved ro-vibrational transitions in C_60_ fullerene, based on the combination of BGC with cavity-enhanced direct frequency comb spectroscopy^16^. Equally noteworthy is the achievement of sub-Hz-precision differential spectroscopy at frequencies near 15 GHz (9.7 × 10^−11^ in fractional terms) between buffer-gas-cooled enantiopure samples of (R)- and (S)- 1,2-propanediol, by means of a tunable microwave Fabry–Perot cavity^17^.

Concerning diatomic or simple polyatomic molecules, several extensive spectroscopic works exist in the literature, with particular relevance to laser cooling and trapping. Here, the main purpose is the determination of vibronic branching ratios to the 10^−5^ level, which is essentially accomplished by application of laser-induced fluorescence (LIF) on cryogenic buffer gas beams^18^. For example, this produced accurate characterization of the *B*^2^Σ^+^ state of BaH^19^, and of the *X*^2^Σ_1/2_ and *A*^2^Π_1/2_ states in CaOH and SrOH^20^. Application of LIF or absorption spectroscopy to cryogenic buffer-gas cooled samples has also resulted in accurate transition frequency measurements. For instance, hyperfine-resolved spectra were obtained for the ${b}^{3}{{{\Sigma }}}^{+},\nu {\prime}=0\leftarrow {X}^{1}{{{\Sigma }}}^{+},\nu {\prime}{\prime}=1$ and ${b}^{3}{{{\Sigma }}}^{+},\nu {\prime}=0\leftarrow {a}^{3}{{\Pi }},\nu {\prime}{\prime}=0$ bands of AlF^21^ and for the ${\tilde{A}}^{2}{{{\Pi }}}_{1/2}(0,0,0)-{\tilde{X}}^{2}{{{\Sigma }}}^{+}(0,0,0)$ band of ^171,173^YbOH^22^, yielding fine- and hyperfine structure parameters with a precision of a few MHz. Similarly, the [557] − *X*^2^Σ^+^(*ν* = 0, 1, 2, 3) and the [561] − *X*^2^Σ^+^(*ν* = 2) visible electronic transitions of YbF were recorded at a near-natural linewidth spectral resolution; particularly, the ^174^YbF isotopologue spectral features were analyzed to produce a new set of spectroscopic parameters for the *X*^2^Σ^+^(*ν* = 2), *X*^2^Σ^+^(*ν* = 3), [557], and [561] states^23^.

Notwithstanding the numerous applications discussed above, the enormous potential offered by buffer-gas-cooled samples for absolute frequency metrology of ro-vibrational spectra has not been fully exploited yet, mainly due to the difficulty in entirely transferring highly developed spectroscopic methods to complex apparatuses involving cryostats. In particular, implementation of effective cavity-enhanced interrogation schemes is restrained by the vibrations originating from the pulse-tube cryocooler which, via the vacuum chamber, couple to the resonator and deteriorate its mechanical stability^24^. In a previous pioneering experiment, we partially overcame this drawback by using specially contrived damping mounts for the high-reflectivity (HR) cavity mirrors, thus succeeding in extending to the cold-temperature range the early proven association of cavity-ring-down (CRD) spectroscopy with sub-Doppler saturation measurements^25,26^. In that case, a statistical uncertainty of 12 kHz (6 × 10^−11^ in fractional terms) was achieved for the frequency measurement of the target transition^27^.

In the present work, we improve this result by one order of magnitude, through an original opto-mechanical design that allows the BGC source to be efficiently coupled to a cavity with finesse as high as 95,000 (HFC). On this basis, we realize an optical-frequency-comb(OFC)-assisted, Pound-Drever-Hall(PDH)-locked CRD spectrometer^28^, additionally making use of two orthogonally polarized laser beams, one for PDH-locking and the other for probing the molecular sample^29–31^. This offers the great advantage of a fast acquisition of cavity-decay events without interrupting the PDH locking, in turn enabling the implementation of a profitable Lamb-dip saturated-absorption cavity ring-down (SCAR) scheme, just recently demonstrated at room temperature by our group^32^. In this configuration, by way of example, we address the acetylene (*ν*_1_ + *ν*_3_) R(1)e ro-vibrational line at 6561.0941 cm^−1^ in a 20-K cell specimen, demonstrating absolute line-center frequency measurements with an overall (statistical + systematic) uncertainty as low as 1.2 kHz (6 × 10^−12^ in fractional terms). In fact, by ranking with the highest accuracy levels obtained so far with deep-rooted methodologies involving room-temperature cell samples, either in the Doppler-limited^33,34^ or Doppler-free regime^35–40^, our achievement finally opens a metrological perspective to the spectroscopy of buffer-gas-cooled molecules.

## Results

### Experimental apparatus

**Fig. 1:**
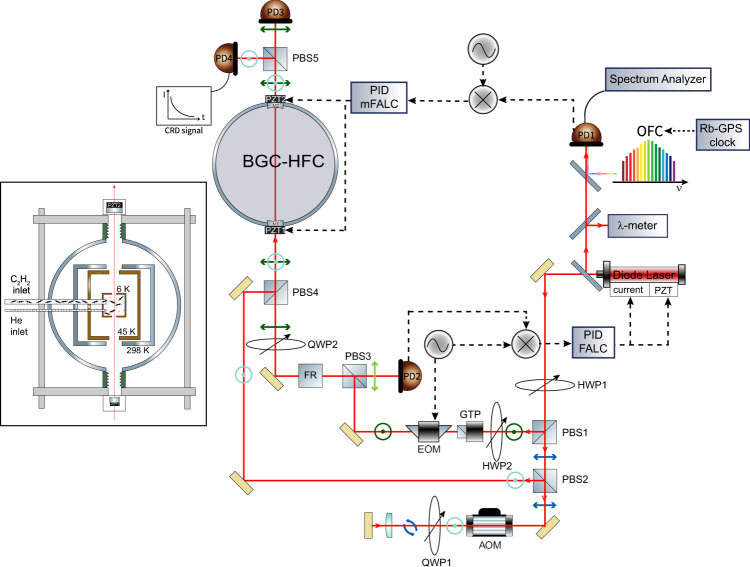
Experimental setup. MAIN FRAME: sketch of the experimental setup for Lamb-dip SCAR spectroscopy of buffer-gas-cooled molecular samples. INSET: zoomed internal (sectional) view of the BGC chamber. An original opto-mechanical design allows the BGC source to be efficiently coupled to a high-finesse cavity; on this basis, an OFC-assisted, PDH-locked spectrometer with crossed polarizations is realized for Lamb-dip SCAR measurements. The following legend holds: HWP half wave-plate, QWP quarter wave-plate, PD photo-detector, PBS polarizing beam splitter, GTP Glan-Taylor polarizer, FR Faraday rotator; AOM acousto-optic modulator, EOM electro-optic modulator.

The experimental apparatus as a whole is shown in Fig. 1. Described in detail in a previous paper^41^, the heart of the BGC source consists of a two-stage pulse tube (PT) cryocooler (Cryomech, PT415) housed in a stainless-steel (S-S) vacuum vessel. In particular, the second stage yields a temperature around 5 K provided that its heat load is kept below 1.5 W; for this purpose, each PT plate is enclosed in a gold-plated shield (equipped with accesses for the passage of the laser beam) in order to suppress black-body radiation. A single S-S pipe, thermally insulated from both the cryocooler stages, is used to introduce both acetylene and helium (contained in room-temperature bottles) into the BGC cell. The injected flux for each gas (*f*_Ac_ and *f*_He_), here expressed in Standard Cubic Centimeters per Minute (1 SCCM = 4.5 ⋅ 10^17^ particle/s), is regulated independently upstream by a 1-%-accuracy flow controller. The BGC cell is a copper cube of side length *L* = 40 mm, in contact with the second PT plate, equipped with two facing circular holes (5 mm diameter) aligned along the axis of the high-finesse spectroscopic cavity. In this configuration, C_2_H_2_ molecules are cooled down to 18 K through multiple collisions with the He buffer gas.

We now come to the delicate point of how to minimize the deleterious effects of the vibrations from the cryocooler on the constituent elements of the high-finesse cavity (HFC), when this is integrated into the BGC source, going to form what from here on we will call the BGC-HFC. The mechanical noise is essentially dominated by the low-frequency components driven by the compression cycle, but high-order harmonics are also present due to distortion modes of the PT tubes and smaller parts of the system with higher resonant frequencies. To address this issue, we act on two fronts.

On one side, there is a careful mechanical arrangement which can be schematically described as follows. The two BGC-HFC mirrors (3 m radius of curvature) are housed in the aforementioned damping mounts at a distance *d* = 98 cm from each other, corresponding to a free spectral range (FSR) of ≃ 150 MHz. Additionally, the mirror mounts are mechanically isolated from the cryostat dewar by a system of edge-welded bellows forming the vacuum connection, and are incorporated in as many massive S-S bars, each resting on its own optical bench by means of damping material (Vulcuren) feet. Finally, two S-S rods, girdling the cryostat dewar, connect the two bars, thus fixing the gross cavity length. Additional Vulcuren damping cylinders are placed in the points of the apparatus most stressed by vibrations.

**Fig. 2:**
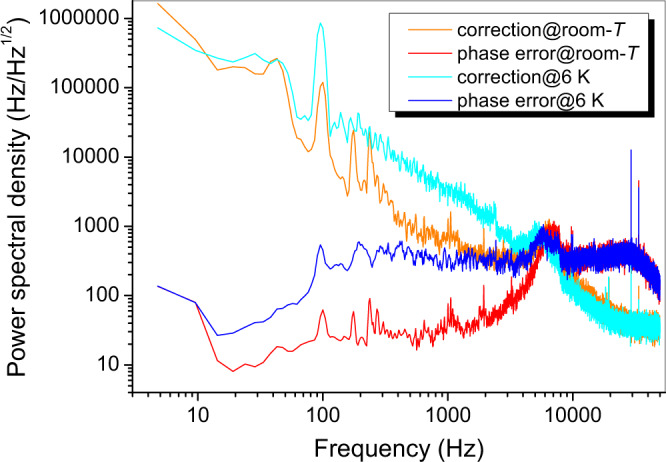
Characterization of the overall frequency reference chain. Characterization of the ECDL → BGC-HFC → OFCS → Rb/GPS lock chain, as derived by the fast Fourier transform (FFT) of the servo (mFALC110) error/correction signal. It is evident the excess noise due to the PT operation (fairly mitigated by the introduction of the Vulcuren elements), which in particular amplifies a structural (i.e., already present at room temperature) resonance at 100 Hz together with its second harmonic.

On the other side, a sophisticated SCAR interrogation scheme is implemented. The radiation source is a cw single-mode external-cavity diode laser (ECDL) emitting around 30 mW in the 1470–1560 nm wavelength interval. The main fraction of the ECDL output beam is divided into the s- and p-polarized radiation, which are separately directed to the BGC-HFC for the PDH locking and the SCAR measurements, respectively. After being phase-modulated, the s-polarized beam is sent to the cavity via an optical circulator. In this way, the light reflected off the cavity is conveyed to an InGaAs photodetector (PD2) in order to generate the PDH error signal, which is used to continuously lock the laser frequency to a resonance of the cavity. The p-polarized component (SCAR beam) double-passes through an acousto-optic modulator (AOM) in cat’s eye configuration^42^, driven at *ν*_AOM_ = 150 MHz, emerging shifted by 300 MHz (i.e., twice the cavity FSR) with respect to the PDH beam. This ensures the cavity resonance condition for the SCAR beam as well, while avoiding interference with the PDH radiation. Then, the PDH (≃120 μW) and SCAR beam combine together through a polarizing beam splitter, before entering the BGC-HFC. Emerging from the latter, the transmitted PDH and SCAR beam are again separated, and eventually collected by two identical photo-detectors (PD3 and PD4, respectively). The actual extinction factor is below 10^−3^, as determined by the ratio of the voltages measured by PD3 and PD4, keeping only the SCAR beam on. Moreover, in order to control the BGC-HFC length, while simultaneously providing absolute frequency calibration of the spectra, the beat note between the ECDL and the *N*-th tooth of an OFC is phase-locked to a local oscillator (*ν*_LO_ = 30 MHz). The free-running and in-loop phase noise of the overall lock chain (ECDL → BGC-HFC → OFCS → Rb/GPS) are shown in Fig. 2, both at room and cryogenic (6 K) temperature, pointing out a total bandwidth of around 9 kHz. In the end, the residual laser linewidth actually entering the final spectroscopic measurement is substantially set by the width of the comb tooth (less than 100 kHz).

In the above configuration, the absolute frequency of the SCAR beam interacting with the cold sample reads as: *ν*_SCAR_ = 2 *ν*_AOM_ + *ν*_CEO_ + *N* *ν*_RR_ + *ν*_LO_, where *ν*_CEO_ (20 MHz) and *ν*_RR_ (250 MHz) are the OFC carrier-envelope offset and mode spacing, respectively. Thus, tuning across the target molecular transition (centered at *ν*_0_) is accomplished by varying *ν*_RR_ in discrete steps. For each *ν*_SCAR_ value, under the continuous PDH locking, the SCAR beam is abruptly switched on/off by the AOM (automatically triggered by a digital delay generator), and the resulting ring-down events are recorded by a 18-bit (vertical resolution) oscilloscope (NI, PXI-5922). Then, based on the theoretical model developed specifically for the Lamb-dip regime in a previous work^32^, the average of 600 acquisitions (each lasting about four times *τ*_0_) is fitted by the function

1 $${{{{{{{\mathcal{W}}}}}}}}(t)={{{{{{{\mathcal{B}}}}}}}}+{{{{{{{\mathcal{A}}}}}}}}\,{e}^{-{\gamma }_{c}t}f[t;{\gamma }_{c},{\gamma }_{g},{U}_{g}({\nu }_{{{{{{{{\rm{SCAR}}}}}}}}}-{\nu }_{0})],$$

with the equation

2 $$f=-{\gamma }_{g}\,\frac{2}{1+\sqrt{1+{e}^{-{\gamma }_{c}t}f(t)\,{U}_{g}({\nu }_{{{{{{{{\rm{SCAR}}}}}}}}}-{\nu }_{0})}}\,f(t)$$

being numerically integrated in the fitting routine (*f*(0) = 1). In the above equations, ${{{{{{{\mathcal{A}}}}}}}}$ and ${{{{{{{\mathcal{B}}}}}}}}$ are the signal amplitude and background, respectively, *γ*_*c*_ ≡ 1/*τ*_0_ is the empty-cavity decay rate, *γ*_*g*_ denotes the decay rate due to the linear gas absorption, and ${U}_{g}({\nu }_{{{{{{{{\rm{SCAR}}}}}}}}}-{\nu }_{0})=S\,[1+{{{{{{{\mathcal{D}}}}}}}}({\nu }_{{{{{{{{\rm{SCAR}}}}}}}}}-{\nu }_{0})]$ represents the dip saturation profile, with *S* = *I*_cav,0_/*I*_sat_ (i.e., the ratio of the intra-cavity radiation intensity to the saturation intensity of the investigated transition) and ${{{{{{{\mathcal{D}}}}}}}}$ being a lineshape function that is equal to 1 in the center and decreases to zero as the detuning increases in either direction. A typical scan across the Lamb-dip profile consists of 56 points spaced on average 180 kHz apart, and takes approximately 1 min (the maximum acquisition rate for ring-down events is 800 Hz). The ultimate spectral feature is the average over 50 single Lamb-dip profiles.

### Spectroscopic data and analysis

**Fig. 3:**
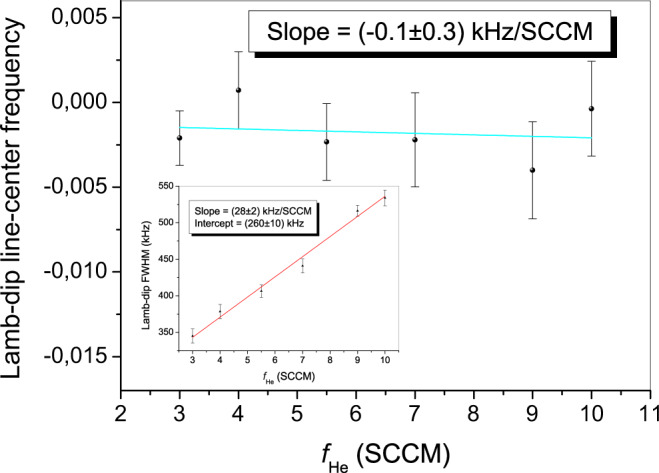
Lamb-dip center frequency vs helium flux. Line-center frequency (main frame) and FWHM (inset) of the saturation dip as a function of *f*_He_, for *f*_Ac_ = 3 SCCM and *P*_SCAR_ = 40 *μ*W. The error bars represent the statistical uncertainty (1-standard deviation) from the Lorentzian fit to the dip saturation profile.

With the ultimate aim of minimizing the uncertainty on *ν*_0_, Lamb-dip spectra (with corresponding underlying Doppler-limited profiles) are initially acquired as a function of *f*_He_, for the lowest He flux and SCAR beam power (*f*_Ac_ = 3 SCCM and *P*_SCAR_ ≃ 40 μW). For a coupling factor *C* ≃ 0.9 and a laser beam waist radius *w*_0_ ≃ 730 μm, this corresponds to an intra-cavity radiation intensity as high as ${I}_{{{{{{{{\rm{cav}}}}}}}},0}=(C{P}_{{{{{{{{\rm{SCAR}}}}}}}}}{{{{{{{\mathcal{F}}}}}}}})/({\pi }^{2}{w}_{0}^{2})\simeq 60$ W/cm^2^. The values extracted for *ν*_0_ and the full width at half maximum (Γ_*L*_) by a Lorentzian fit to the dip saturation profiles are plotted in Fig. 3. In particular, the slope of a linear fit to the Γ_*L*_(*f*_He_) data points provides the (low-temperature) foreign collisional-broadening coefficient, *γ*_He_ = (28 ± 3) kHz/SCCM; on the contrary, the collisional shift coefficient is not significantly estimated: *δ*_He_ = (−0.1 ± 0.3) kHz/SCCM. In order to provide the above coefficients in more commonly used units, we denote by *N*_in_ (*N*_out_) the number of helium atoms injected (emerging) into (from) the BGC cell. Then, in stationary conditions, we have ${f}_{{{{{{{{\rm{He}}}}}}}}}\equiv d{N}_{{{{{{{{\rm{in}}}}}}}}}/dt=d{N}_{{{{{{{{\rm{out}}}}}}}}}/dt=({n}_{He}\,{\overline{v}}_{{{{{{{{\rm{He}}}}}}}}}\,{A}_{{{{{{{{\rm{ape}}}}}}}}})/4$, where *n*_He_ and ${\overline{v}}_{{{{{{{{\rm{He}}}}}}}}}=\sqrt{8{k}_{B}T/(\pi {m}_{{{{{{{{\rm{He}}}}}}}}})}$ (with *m*_He_ being the helium mass) are the steady-state density and the mean thermal velocity of the gas atoms inside the cell, respectively, and *A*_ape_ is the total (for both exit holes) aperture area^43^. Finally, the conversion factor ${n}_{{{{{{{{\rm{He}}}}}}}}}/{f}_{{{{{{{{\rm{He}}}}}}}}}=4/({\overline{v}}_{{{{{{{{\rm{He}}}}}}}}}{A}_{{{{{{{{\rm{ape}}}}}}}}})$ is found, which allows to rewrite *γ*_He_ = (2.4 ± 0.3) cm^−1^/atm and *δ*_He_ = (−0.01 ± 0.03) cm^−1^/atm. A similar analysis is repeated as a function of *f*_Ac_ (*f*_He_ = 3 SCCM, *P*_SCAR_ ≃ 40 μW), in order to determine the self collisional-broadening coefficient, *γ*_Ac_ = (30 ± 5) kHz/SCCM, and the corresponding shift coefficient, *δ*_Ac_ = (−0.2 ± 0.4) kHz/SCCM. In this case, however, a real conversion between SCCM and atm units cannot be done. Indeed, contrary to the case of helium, many of the C_2_H_2_ molecules injected into the cell freeze upon impact on the walls (whose temperature is well below the acetylene melting point). Therefore, the acetylene density inside the cell depends not only on the injected C_2_H_2_ flux, but also on the helium density, as the random walk experienced by the molecules in the He bath increases the diffusion time (i.e., the lapse between entering the cell and sticking on its walls). Ultimately, even with the same *f*_Ac_ value, very different molecular densities can occur, depending on the amount of helium present.

**Fig. 4:**
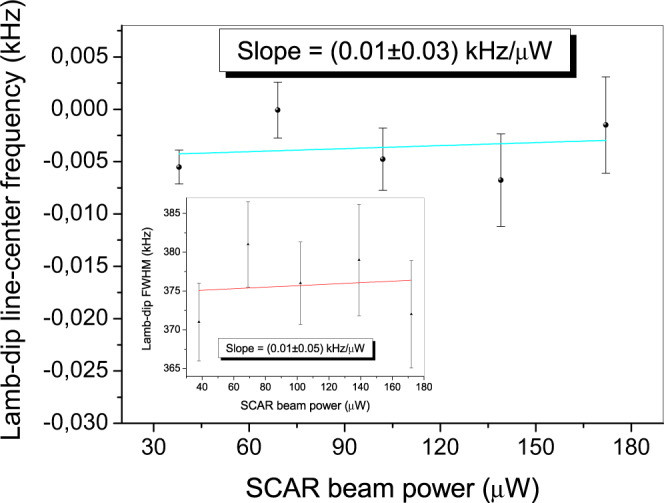
Lamb-dip center frequency vs SCAR beam power. Line-center frequency (main frame) and FWHM (inset) of the saturation dip as a function of *P*_SCAR_, for *f*_He_ = *f*_Ac_ = 3 SCCM. The error bars represent the statistical uncertainty (1-standard deviation) from the Lorentzian fit to the dip saturation profile.

Finally, as shown in Fig. 4, we investigate the behavior of the line-center frequency as a function of *P*_SCAR_ (*f*_Ac_ = *f*_He_ = 3 SCCM), finding a power shift coefficient of *δ*_*P*_ = (0.01 ± 0.03) kHz/μW; as illustrated in the corresponding inset, the SCAR analysis leaves saturation broadening effects out of the widths in the *U*_*g*_ profiles^32^.

**Fig. 5:**
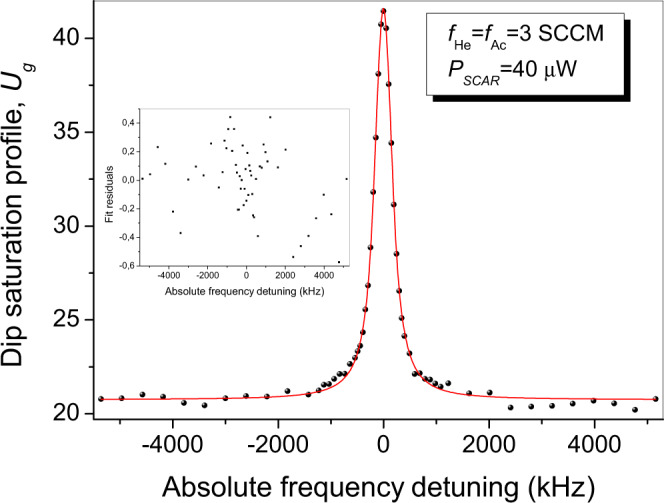
Typical Lamb-dip absorption profile. Lamb-dip profile (with corresponding fit residuals) recorded for *f*_Ac_ = *f*_He_ = 3 SCCM and *P*_SCAR_ = 40 μW, exhibiting the highest SNR (90) and narrowest FWHM (370 kHz).

As displayed in Fig. 5, the choice *f*_Ac_ = *f*_He_ = 3 SCCM with *P*_SCAR_ = 40 μW corresponds to the highest SNR (≃90) and narrowest linewidth, while simultaneously minimizing the molecular sample temperature: *T* = (20 ± 2) K, as measured by the width $\sigma=({\nu }_{0}/c)\sqrt{8\ln 2{M}^{-1}{k}_{B}T}$ of the Gaussian profile in which the dip is dug (with *M* being the acetylene mass). The observed width, Γ_3,3_ = (370 ± 5) kHz, is consistent with the combination of the three main broadening contributions: the collisional term *γ*_coll_ = (170 ± 30) kHz, the residual laser emission linewidth *γ*_las_ ≃ 100 kHz, and the transit-time ${\gamma }_{{{{{{{{\rm{tt}}}}}}}}}=\sqrt{16\ln 2/{\pi }^{3}}\,{w}_{0}^{-1}\sqrt{({k}_{B}T)/M}\simeq 70$ kHz (referring to a single molecule traveling with the mean thermal speed of the ensemble). Specifically, after adding the first two Lorentzian contributions (both of the homogeneous type), and then taking the Voigt convolution with the remaining (inhomogeneous-type) transit-time term, one gets the expected final dip width: $0.5346\cdot ({\gamma }_{{{{{{{{\rm{coll}}}}}}}}}+{\gamma }_{{{{{{{{\rm{las}}}}}}}}})+\sqrt{{\gamma }_{{{{{{{{\rm{tt}}}}}}}}}^{2}+0.2166\cdot {({\gamma }_{{{{{{{{\rm{coll}}}}}}}}}+{\gamma }_{{{{{{{{\rm{las}}}}}}}}})}^{2}}\simeq 320$ kHz (Olivero’s formula^44^).

Concerning the determination of the absolute line-center frequency, a statistical uncertainty as low as 1.1 kHz is found from the Lorentzian lineshape fit, while the relevant sources of systematic uncertainty are listed in Table 1. On the time scale typical of our spectroscopic acquisitions, the stability of the GPS-based reference chain contributes with an uncertainty of 0.5 kHz (the contribution related to the AOM driving frequency is only at the Hz-level). The accuracy of the flow meters (1%) translates into an overall collisional shift uncertainty of 0.03 SCCM × (0.3 + 0.4) kHz/SCCM = 0.02 kHz. Moreover, while the recoil shift is canceled out in Lamb-dip spectroscopy, the second-order Doppler shift is estimated as *ν*_0_*k*_*B*_*T*/(*Mc*^2^) = 0.02 kHz. Also, a 15% uncertainty on the SCAR beam power gives a contribution of (40 ⋅ 0.15) μW × 0.03 *kHz*/μW = 0.18 kHz. Finally, by allowing the static width of the Lorentzian lineshape to vary sigmoidally, an uncertainty around 70 Hz is assigned to asymmetries in the Lamb-dip fit. By adding in quadrature all the terms, the overall (type A + type B) 1*σ* uncertainty amounts to 1.2 kHz (6 × 10^−12^ in fractional terms): *ν*_0_ = (196 696 652 914.3 ± 1.2) kHz. Our value is consistent with the most accurate (room-temperature) determination so far: (196 696 652 918 ± 2) kHz, by ref. 45.

**Table 1:** Summary of major uncertainties associated with the absolute determination of the center frequency of the C_2_H_2_(*ν*_1_ + *ν*_3_) R(1)e ro-vibrational transition, for *P*_SCAR_ = 40 *μ*W and *f*_Ac_ = *f*_He_ = 3 SCCM

Contribution	Uncertainty (kHz)
Statistical	1.1
Foreign collisional shift	0.01
Self collisional shift	0.01
Power shift	0.2
GPS-based reference chain	0.5
Second-order Doppler shift	0.02
Lamb-dip profile fit	0.07
Total	1.2 (6 ⋅ 10^**−12**^)

## Discussion

In conclusion, by applying the Lamb-dip SCAR technique to a buffer-gas-cooled molecular sample, we have measured the absolute frequency of a given ro-vibrational transition with an overall relative uncertainty of 6 × 10^−12^. Except for selected lines of exclusive molecules (SF_6_, OsO_4_, CH_4_) which, thanks to a long course of spectroscopic research on secondary frequency standards, are known with accuracy below 10^−13^^46–49^, our achievement is in line with the state of the art on room-temperature cell samples. Therefore, being straightforwardly extendable to many species in different spectral regions, our approach establishes a complementary frequency-metrology tool, especially useful for those molecules (even simple ones) whose room-temperature spectrum is congested. In this regard, it should be emphasized that acetylene exhibits pretty isolated lines already at room temperature, especially in the Lamb-dip regime. Thus, the effects of the expected BGC-induced spectral decongestion are not directly demonstrated here, and a franker experimental proof implies moving to a more complex molecular species. Indeed, we are planning a high-resolution spectroscopy experiment on buffer-gas-cooled fluoroform (CF_3_H), in order to excite a predicted two-photon transition at around 8.6 μm wavelength^50^, so far not identified due to the presence of numerous concurrent lines^51^.

Moving to the field of ultracold molecular gases, our result also fairly compares to the records in the low 10^−12^ range so far attained with trapped ions^4,52^ or photo-associated dimers (e.g., Sr_2_) in optical lattices^53^, in spite of the vastly different linewidth observed for the spectroscopic feature: kHz/sub-KHz in the former case, a few tens of Hz in the latter case. Therefore, already at the current level, our method represents a valuable option for frequency metrology of cold spectra in a different low-temperature range, on a much wider class of molecules.

At the same time, our novel approach still has ample room for improvement, laying the groundwork for approaching the 10^−13^ accuracy range. Relevant upgrades could include the prestabilization of the probe laser against an ultra-low-expansion (ULE) reference cavity, as well as the implementation of even more sophisticated spectroscopic techniques, such as 2-photon cavity-ring-down spectroscopy^54^ or noise-immune cavity-enhanced optical-heterodyne molecular spectroscopy (NICE-OHMS)^2,55,56^. Accordingly, the Rb-GPS standard should be replaced with the clock laser delivered from Istituto Nazionale di Ricerca Metrologica (INRIM) through the National Optical Fiber Link (already fully operational in our lab and routinely referenced to the primary Cs fountain^57^). Definitely, a big step forward will be moving from a static cryogenic molecular sample to a buffer-gas-cooled beam (high brilliance, slow laboratory-frame velocity, and collision-free environment), until reaching the observation of two-photon Ramsey fringes in the spatial domain^9^.

Ultimately, this will trigger a new generation of low-temperature precision spectroscopy studies, of relevance both for the aforementioned fundamental Physics researches and for the identification of more efficient laser cooling schemes toward the achievement of quantum degeneracy in molecular systems^58^.

## Methods

### Optical setup

The BGC-HFC input mirror (M1) has a 0.25-inch diameter (with a thickness of 4 mm) and reflectivity > 99.97%, while the second mirror (M2) has a 1-inch diameter and reflectivity > 99.995%. The measured empty-cavity decay time is *τ*_0_ ≃ 42*μ*s, corresponding to a finesse ${{{{{{{\mathcal{F}}}}}}}}=(\pi c{\tau }_{0})/d\simeq 95000$. M1 and M2 are mounted on annular PZTs, PZT1 (HPSt150/20-15/12, Piezomechanik) and PZT2 (PD120.31, Round PICMA), respectively. The s-polarized beam emerging from PBS1 first passes through a Glan-Taylor calcite polarizer (GTP), with a vertical transmission axis, providing an extinction ratio of ≃100 000. Afterward, the PDH beam is frequency modulated by a temperature-controlled electro-optic phase modulator (EOM) forming sidebands at 30 MHz relative to the carrier, and then enters an optical circulator, consisting of PBS3 and a Faraday rotator (FR). The generated PDH error signal is processed by a proportional-integral-derivative (PID) servo (Toptica Photonics, FALC 110), which provides external correction to the ECDL frequency, acting both on the piezo-electric transducer (PZT) of the external cavity (100 Hz bandwidth) and on the laser current driver (1 MHz bandwidth).

The beat note between the ECDL and the OFC (Menlo Systems, FC-1500-250-WG) is detected by a PIN-InGaAs fast photodiode (PID1) and, after proper band-pass filtering (to minimize noise contribution from other comb lines) and amplification, processed by an electronic servo (Toptica Photonics, mFALC110), whose correction is carried out via a slow feedback to PZT1, plus a fast one to PZT2. In turn, both *ν*_CEO_ and *ν*_RR_ are stabilized against a Rb/GPS clock, providing an accuracy of 10^−13^ and a fractional stability (Allan deviation) between 4 × 10^−13^ and 8 × 10^−13^ for an integration time between 10 and 1000 s.

## Data Availability

All final data generated in this study are available within the article. Other material that supports the findings is available from the corresponding author on request.
